# Central venous catheter cannulation by a trained ultrasound team in onco-haematological patients

**DOI:** 10.1186/2197-425X-3-S1-A78

**Published:** 2015-10-01

**Authors:** JA Soler, MD Casado, SM Botías, F Caballero, MB Almaida, M Fernández, G Quintanilla, I Heras, A Carrillo

**Affiliations:** Intensive Care Unit, Morales Meseguer Hospital, Murcia, Spain; Hematology Department, Morales Meseguer Hospital, Murcia, Spain

## Introduction

Central venous catheters (CVC) frequently are needed in onco-haematological patients during their hospitalization. In this particular population, local complications related to cannulation, such us bleeding or hematoma, are increased. Nowadays, ultrasound guided cannulation can provide benefits to avoid these adverse events.

## Objectives

To analyze safety and effectiveness of ultrasound guided (USG) CVC cannulation performed by a trained team in onco-haematological patients.

## Methods

Prospective 6 months pre-post study of all CVC cannulations, except those peripherally inserted, in patients from onco-haematological ward in a university teaching hospital. Ultrasound team was composed by 3 physicians and 2 nurses trained on vascular USG cannulation. During “pre-team” period, CVC cannulation was performed by the intensivist on duty using anatomical landmark or USG technique. In the second period, CVC cannulation was exclusively performed by USG team. Demographic and clinical data as well as variables related to cannulation were collected. Results are expressed as mean ± standard deviation and percentages. Comparisons between variables were performed by Student´s t-test and Pearson´s chi-squared test or Fisher's exact test.

## Results

A total of fifty seven CVC cannulations were performed in forty two patients. Thirty one CVC cannulations (54.4%) were performed by USG team. No differences were observed between post and pre-team period regarding sex (women 35.5% vs. 30.8%; p = 0.71), age (54.4 ± 16.2 years vs. 51.7 ± 18.0 years; p = 0.56), catheter indication (chemotherapy: 77.4% vs. 73.1%; p = 0.76; apheresis: 12.9% vs. 19.2%; p = 0.72; medical treatment: 3.2% vs. 7.7%; p = 0.59; parenteral nutrition: 6.5% vs. 0.0%; p = 0.50) or placement (right internal jugular: 64.5% vs. 73.1%; p = 0.57; left internal jugular: 22.6% vs. 7.7% p = 0.16; right femoral: 12.9% vs. 19.2%; p = 0.72). Success on first attempt was higher in USG team period (87.1% vs. 57.7% p = 0.02). In the same way, there was a lower rate of hematoma and bleeding at 24 hours in the second period (12.9% vs. 38.5%; p = 0.03) with no differences in platelet account (124.9x10^3^ ± 91.9x10^3^ vs. 131.5x10^3^ ± 73.0x10^3^; p = 0.78), coagulation parameters (INR: 1.1 ± 0.3 vs. 1.0 ± 0.2; p = 0.41; TTPa: 24.6 ± 8.9 seconds vs. 25.4 ± 9.7 seconds; p = 0.75) or need for transfusion (26.7% vs. 16.0%; p = 0.51). No severe complications were observed in both periods.

## Conclusions

Ultrasound guided CVC cannulations performed by a trained team is a safe and effective procedure in onco-haematological patients. This approach is also related to a lower rate of local complications.

